# Intentions of hospital pharmacists to use digital technology in their daily practice: a cross-sectional survey using the Theory of Planned Behaviour

**DOI:** 10.1007/s11096-025-01868-5

**Published:** 2025-02-10

**Authors:** Kamer Tecen-Yucel, Nesligül Ozdemir-Ayduran, Emre Kara, Kutay Demirkan, Betul Okuyan

**Affiliations:** 1 Department of Clinical Pharmacy, Faculty of Pharmacy, Anadolu University, Eskisehir, Türkiye; 2 Department of Clinical Pharmacy, Faculty of Pharmacy, Inönü University, Malatya, Türkiye; 3 Department of Clinical Pharmacy, Faculty of Pharmacy, Hacettepe University, Ankara, Türkiye; 4 Department of Clinical Pharmacy, Faculty of Pharmacy, Marmara University, Istanbul, Türkiye

**Keywords:** Digital health, Digital technology, Hospital pharmacists, The theory of planned behaviour

## Abstract

**Background:**

Digital technology has been widely integrated into healthcare. This encompasses knowledge, skills, and practices related to the development and use of health technologies. The behavior of health professionals is critical to the adoption of these technologies.

**Aim:**

This study aimed to investigate factors associated with hospital pharmacists’ intention to use digital technology in their daily practice using the Theory of Planned Behaviour (TPB).

**Method:**

In this cross-sectional study, a paper-based survey was conducted among hospital pharmacists who attended the National Hospital Pharmacy Congress in Türkiye in March 2022. A valid and reliable Turkish scale based on the TBP was used to identify factors associated with the intention score by a multiple linear regression model.

**Results:**

One hundred ten participants completed the survey (response rate: 44.0%). Seventy percent of pharmacists reported that they had not received prior training in digital technologies. More than eighty percent of the participants said they intend to use digital technology in daily practice. The higher scores of attitudes (*p* = 0.005), self-efficacy (*p* < 0.001), and working place (*p* = 0.017) were associated with increased intention scores.

**Conclusion:**

Positive attitudes, higher self-efficacy, and working in tertiary hospitals were associated with hospital pharmacists’ intentions to use digital technology in daily practice. These factors should be considered in developing interventions to promote digital technology use of hospital pharmacists.

## Impact statements

- The theory of planned behaviour model could be used to develop interventions to promote the use of technology by hospital pharmacists.
- Higher self-efficacy and positive attitudes of hospital pharmacists were associated with their intentions to use digital technologies in their daily practice.
- Working in tertiary hospitals was also related to their daily intention to use digital technology.

## Introduction

Medication-related errors comprise between 5 and 41% of all hospital admissions worldwide [1]. According to a recent published systematic review, one fourth of hospitalized older patients had at least one adverse drug reaction [2]. According to the World Health Organization (WHO), digital technology in the healthcare system can be defined as: “the field of knowledge and practice associated with the development and use of digital technologies to improve health. Digital health expands the concept of eHealth to include digital consumers, with a wider range of smart and connected devices.” In the use of digital technology in the healthcare system, individual barriers such as the user’s level of knowledge and usage skills are important [3]. Digital technology is needed to reduce the harm caused by repeated errors in the healthcare system and to promote the safe use of medicines. However, it should not be forgotten that while digital technology can reduce harm caused by certain errors, the likelihood of new types of errors arising from the use of these technologies is also quite high. Since medication safety is complex, digital technologies are only one solution [4].

The last two decades have seen the integration of digital technology into healthcare through the evolution of information technology [5]. The positive and negative effects of developments in digital technology, including their impact on human health, have been and will continue to be the focus of active speculation. However, evidence suggests that advancements including cloud computing, blockchain, machine learning, artificial intelligence, digitally mediated diagnostics and treatment, telehealth, and consumer-focused mobile health applications are now commonly employed in biomedical sciences, healthcare services, and self-management. These advancements are anticipated to facilitate earlier diagnoses and treatments, improve outcomes, and promote increased patient engagement [6].

Pharmacists show interest in using digital technology in their routine practice as healthcare professionals. Reports indicate the utilization of digital technologies for delivering clinical pharmacy services, including medication review, health education, and chronic illness management [7, 8]. Telemedicine and digital technology applications have recently gained popularity in the pharmacy environment [9].

The digitalization of healthcare offers many opportunities to improve individual patients’ understanding and self-management of prescribed treatments. The European Association of Hospital Pharmacists’ (EAHP) 2017 position paper on eHealth and mHealth recognizes the need for adequate oversight of mobile solutions to minimize the possible negative impact of giving patients inconsistent, inaccurate, or low-quality advice. Dosage advice and dosage calculators are two areas where this could be a severe issue. EAHP promotes the hospital pharmacist’s unique position as a bridge between medicines and technology in advising patients on the proper use of medications [10]. The adoption of digital technologies by hospital pharmacists in their pharmacy services has the potential to be a mitigating factor in medication safety issues. Identifying the inhibitors and enablers influencing pharmacists’ behaviour is essential to improving digital technology adoption among hospital pharmacists. Despite the considerable benefits that virtual-based digital health technologies can offer patient care, there are a variety of barriers to overcome, ranging from data security to healthcare professional adoption [11]. A systematic evaluation analyzing the technological adoption among healthcare professionals analyzed 142 studies. The findings revealed that innovativeness, computer self-efficacy, anxiety, and trust are the primary elements affecting diverse healthcare technology. The results revealed that Taiwan and the USA are at the top of research concerning technology acceptance in healthcare, with a notable increase in studies centered on electronic medical record systems and telemedicine [12]. In a study conducted in Türkiye, the intention of physicians to use personal digital assistant technology is explained by perceived ease of use and perceived usefulness [13]. In another study conducted in Türkiye about 10 years ago, system factors, perceived ease of use, perceived usefulness, and perceived behavioral control explained almost fifty percent of pharmacists’ intention to use Medula which is a network-based application [14]. However, there is no study in Türkiye that assesses intention to use digital technology in daily practice within the healthcare system without adhering to a specific system.

The Theory of Planned Behaviour (TPB) is commonly used to predict behaviour. The difference between TPB and other alternative models like the Unified Theory of Acceptance and Use of Technology (UTAUT) and Technology Acceptance Model (TAM) is that TPB is used to measure the cognitive and psychological aspects of the technology by the user while the others mostly concentrate on the technology’s capabilities. TPB was considered the most suitable model for our study, which examines hospital pharmacists’ intentions to use digital technology in their daily practice. According to the TPB, subjective norm (a person’s perception of norms in society involving the adoption of specific behaviors), attitude, perceived behaviour, self-efficacy, past behaviour and intention influence the development of behaviour [15, 16]. There are a few studies evaluating hospital pharmacists’ intentions to provide medication therapy management [17] and palliative care management [18] using the TPB model.

### Aim

This study used the TPB to investigate factors associated with hospital pharmacists’ intention to use digital technology in their daily practice in Türkiye.

### Ethics approval

Approval for the study was obtained from the Hacettepe University Health Sciences Research Ethics Committee (GO-22/246), and all participants completed informed consent forms.

## Method

### Study type, setting, and subjects

In this cross-sectional study, a paper-based survey was conducted among hospital pharmacists who attended the National Hospital Pharmacy Congress in Türkiye in March 2022. No incentives were provided to encourage participation.

### Data collection

The survey comprised two parts. The first part encompassed participants’ demographic characteristics (including their sex, age, and educational status), working institution, work experience, and receiving training on the digital health system (including mobile applications, artificial intelligence, device usage, telehealth [tele-medicine], online pharmacy, blockchain technology, digital medicines, digital management systems, information systems), the frequency of using digital health service (daily, weekly, monthly, 1–2 times a year, and never).

A TPB-based scale was administered in the second section. The scale, comprising 37 items, was developed based on TPB literature [19, 20] and a guidebook [21] intended for health services researchers creating TPB studies. The digital technology assessed in the survey is defined as “hardware and software technological products that, with their embedded software, computers, internet, phones, tablets, and information system software, facilitate the services of healthcare professionals and enable patients to receive better services” [22]. The scale has 37 items that assess attitude (ATT; 9 items), subjective norm (SN; 3 items), perceived behavioural control (PBC; 2 items), self-efficacy (SE; 3 items), past behaviour (PB; 9 items), and intention (INT; 11 items). The items were evaluated utilizing a 5-point Likert scale, ranging from strongly disagree (1) to strongly agree (5) for ATT, SN, PBC, SE and INT. For these items; positive/high frequency means the percentage of agreement (total of strongly agreed and agreed), and negative/low frequency means the percentage of disagreement (total of strongly disagree, disagree, and neutral). PB items were scored using a 5-point Likert scale, ranging from never (1) to regularly (5). For this item; positive/high frequency means the percentage of the total of regularly and often; negative/low frequency means the percentage of the total of never, occasionally, and sometimes. The processes of Turkish translation and cultural adaptation followed the guidelines established by the International Society for Pharmacoeconomics and Outcomes Research (ISPOR) [23]. The expert panel was composed of four clinical pharmacists and has evaluated the content validity of the TPB-based scale. The expert panel identified and arranged the items constituting the TPB structure and conducted translation and back-translation for language adaptation. A pilot study involving 20 pharmacists was performed to assess the clarity of all items. At least 12 participants were required for the pilot study [24]. For test–retest analysis, at least 30 pharmacists (not included in the study dataset) were require [25] to fill at baseline and two weeks later. We sent the survey to 40 pharmacists for test–retest analysis. Test–retest was undertaken to assess the reliability of the survey, ensuring that it produces consistent and stable results over time. The time required to complete the scale was about 10 to 15 min.

### Sample size

Using multiple regression analyses for TPB research presumed a minimum low effect size (i.e., multiple R of around 0.3) is appropriate [26]. In general, a sample size of 80 is recommended for TPB-based studies [27].

### Statistical analysis

Data were analyzed using descriptive statistics (including mean, standard deviation, median, interquartile range, frequency, and percentages). The statistical analysis of the study was performed using SPSS version 23 (IBM, Armonk, NY). The normality of the data was determined using Shapiro–Wilk tests. Test–retest reliability was calculated using the intraclass correlation coefficient, expressing complete agreement without taking into account how different questions interact. Exploratory factor analysis (EFA) with varimax rotation was performed in order to determine the underlying structure of the TPB. EFA was essential to verify that the survey items accurately represented the TPB constructs and to ensure the survey’s structure was robust before further statistical analysis, such as reliability testing and regression analysis. The study evaluated the Kaiser–Meyer–Olkin (KMO) Measure of Sampling Adequacy and Bartlett’s Test of Sphericity. The KMO assesses sample adequacy, reflecting the proportion of total variance clarified by underlying factors; values approaching 1 indicate a more suitable sample. Bartlett’s test assesses if the correlation matrix significantly deviates from an identity matrix, hence indicating the suitability of factor analysis. To show the internal consistency of the measures, Cronbach’s alpha was calculated for each construct and the TPB-based scale (if Cronbach’s alpha is > 0.7, it is defined as acceptable; if Cronbach’s alpha is > 0.9, it is defined as excellent) [28]. According to the TPB, univariate analysis was used to identify factors related to their intentions to use digital technology in their daily practice. Based on the univariate analysis, variables with *p* < 0.25 were selected as candidate variables. Each of the 37 items was scored using a 5-point Likert scale (ranging from 1 to 5) in the survey. The final score for each subscale (ATT, SN, SE, PBC, PB and INT) was calculated as the average of the scores obtained for each one of the items in the subscales, and the data were converted into continuous values [29, 30]. The mean of the TPB score ranges from 1 to 5. Following that, the multiple linear regression model was applied with a significance level of *p* < 0.05. Multiple linear regression was conducted to define the effect of each independent variable on pharmacists’ intention to use digital technology and to measure the magnitude of this effect independently of other variables, without ignoring the influence of other factors.

## Results

Among 250 attendees of congress, one hundred ten participants (response rate: 44.0%) completed a paper-based survey. The median (IQR) age of the participants was 32 (10.25). Eighty percent of the participants were women. Thirty-three percent of the participants had a postgraduate education. Most pharmacists (*n* = 76) stated that they had not previously received any training on digital technology. Thirty-four who received training in the field of digital technology had been trained in topics such as mobile applications (*n* = 25), digital management systems (*n* = 17), device use (*n* = 11), online pharmacy (*n* = 8), artificial intelligence (*n* = 5), tele-health (*n* = 3), digital medicine (*n* = 2), information systems (*n* = 2) and blockchain technology (*n* = 1). The characteristics of participants are presented in Table 1. The data were normally distributed (*p* > 0.05).

**Table 1:** Characteristics and professional experiences of participants (*n* = 110)

Variables	*n* (%)
*Sex*	
Female	88 (80.0)
Male	22 (20.0)
*Educational status*	
Bachelor’s degree	73 (66.4)
MSc with thesis	18 (16.4)
MSc without thesis	10 (9.0)
PhD degree	9 (8.2)
*Years of experience in hospital pharmacy *	
< 1	8 (7.3)
1–5	41 (37.3)
6–10	26 (23.6)
> 10	35 (31.8)
*Current working institution*	
Tertiary hospital	74 (67.2)
Secondary care hospital	36 (32.8)
*Digital technology usage frequency*	
Every day	58 (52.7)
Weekly	10 (9.1)
Monthly	19 (17.3)
1–2 times a year	16 (14.5)
Never	7 (6.4)

*MSc* Master of Science, *PhD* Doctor of Philosophy

There were 110 responses from hospital pharmacists that were analyzed. To determine the sufficiency of our sample size, we employed Soper’s [31]. Calculator for Post-Hoc Statistical Power in Multiple Regression. Soper’s calculator [31] demonstrated that our sample size was sufficiently adequate given our five latent variables on the TPB scale, a R^2^ of 0.39, and a sample size of 110 (observed statistical power = 0.99).

The test–retest reliability of the responses from 39 pharmacists (response rate 97.5%) who completed the survey at baseline and again two weeks later was used to determine the intraclass correlation coefficient. The result was 0.80, indicating strong reliability (*p* < 0.001).

A prerequisite test for factor analysis revealed the Kaiser–Meyer–Olkin sample adequacy measure provided a value of 0.85, and Bartlett’s Test of Sphericity indicated a significant difference (*p* < 0.001) between the identity matrix and the questionnaire’s correlation matrix, demonstrating that factor analysis is feasible. The exploratory factor analysis identified six subscales, accounting for 78.2% of the total variation. Almost every item has a significant (> 0.70) loading on its underlying construct, demonstrating convergent validity. Cronbach’s alpha was 0.95 (excellent level of reliability) for the total TBP-based scale. Cronbach’s alpha was 0.93 (excellent level of reliability), 0.73 (acceptable level of reliability), 0.78 (acceptable level of reliability), 0.95 (excellent level of reliability), 0.96 (excellent level of reliability), and 0.98 (excellent level of reliability) for attitude, subjective norm, perceived behavioural control, self-efficacy,past behaviour and intention, respectively. Table 2 shows factor loading values for subscales.

**Table 2:** Survey statements and descriptive statistics (*n* = 156)

Construct	Survey statement	Factor loading	Median (IQR)	Mean ± SD	Positive/high frequency (%)	Negative/low frequency (%)
Attitude				4.65 ± 0.09		
ATT1	The use of digital technology accelerates drug supply to services	0.71	5 (1)	4.47 ± 0.809	98 (89.1)	12 (10.9)
ATT2	Using digital technology helps me provide patient education and counseling	0.78	5 (1)	4.32 ± 0.845	92 (83.6)	18 (16.4)
ATT3	Digital technology helps pharmacists prevent medication errors and improve drug safety	0.68	5 (1)	4.55 ± 0.630	104 (94.5)	6 (5.5)
ATT4	Digital technology supports rational medication use and contributes to optimizing medication efficacy	0.68	5 (1)	4.51 ± 0.660	104 (94.5)	6 (5.5)
ATT5	Using digital technology can reduce medication costs	0.76	5 (1)	4.29 ± 0.850	90 (81.8)	20 (18.2)
ATT6	The use of digital technology can significantly benefit patients’ treatment outcomes	0.79	4 (1)	4.25 ± 0.840	91 (82.7)	19 (17.3)
ATT7	Using digital technology can improve patients’ quality of life	0.85	4 (1)	4.17 ± 0.811	89 (80.9)	21 (19.1)
ATT8	The use of digital technology can increase compliance with the guidelines on disease and medication management	0.68	4 (1)	4.40 ± 0.638	105 (95.5)	5 (4.5)
ATT9	The use of digital technology can increase the professional success of pharmacists	0.70	4 (1)	4.29 ± 0.828	92 (83.6)	18 (16.4)
Subjective norm				3.79 ± 0.184		
SN1	Hospital managers (chief physician/manager) support the integration of digital technology into pharmacy services	0.78	3 (1)	3.35 ± 1.080	52 (47.3)	58 (52.7)
SN2	My chief pharmacist supports my use of digital technology in pharmacy services	0.86	4 (1)	4.0 ± 0.958	87 (79.1)	23 (20.9)
SN3	Other pharmacist colleagues support my use of digital technology in pharmacy services	0.54	4 (1)	4.09 ± 0.657	91 (82.7)	19 (17.3)
Perceived behavioural control				3.85 ± 0.318		
PBC1	I have the necessary knowledge and skills to use digital technology in pharmacy services	0.73	4 (1)	3.69 ± 1.020	73 (66.4)	37 (33.6)
PBC2	I have the necessary hardware support (such as software) to use digital technology in pharmacy services	0.87	3 (2)	3.14 ± 1.071	42 (38.2)	68 (61.8)
Self-efficacy				3.54 ± 0.141		
SE1	It will be easy for me to improve compliance with the underlying guidance on disease and medication management by using digital technology	0.76	4 (1)	4.25 ± 0.670	100 (90.9)	10 (9.1)
SE2	It will be easy for me to avoid drug related problems using digital technology	0.82	4 (1)	4.29 ± 0.640	103 (93.6)	7 (6.4)
SE3	It will be easy for me to detect drug related problems using digital technology	0.81	4 (1)	4.30 ± 0.657	100 (90.9)	10 (9.1)
Past behaviour				3.57 ± 0.323		
PB1	Providing patient education and counseling using digital technology	0.81	3 (2)	2.95 ± 1.464	46 (41.8)	64 (58.2)
PB2	Reducing medication costs using digital technology	0.75	4 (2)	3.19 ± 1.430	55 (50.0)	55 (50.0)
PB3	Monitor and manage medication therapy using digital technology	0.86	4 (2)	3.39 ± 1.447	63 (57.3)	47 (42.7)
PB4	Reducing medication dosing errors using digital technology	0.84	4 (2)	3.65 ± 1.259	71 (64.5)	39 (35.5)
PB5	Performing therapeutic drug monitoring using digital technology	0.88	3 (2)	3.14 ± 1.365	50 (45.5)	60 (54.5)
PB6	Increasing patient medication compliance (treatment compliance) using digital technology	0.92	3 (2)	2.95 ± 1.442	48 (43.6)	62 (56.4)
PB7	Increasing compliance with guidelines for disease and medication management by using digital technology	0.87	4 (3)	3.40 ± 1.409	62 (56.4)	48 (43.6)
PB8	Identifying disease-related risk factors using digital technology	0.89	3 (2)	3.10 ± 1.414	51 (46.4)	59 (53.6)
PB9	Reducing drug side effects using digital technology	0.89	3 (2)	3.09 ± 1.385	50 (45.5)	60 (54.5)
Intention				4.22 ± 0.189		
INT1	I intend to keep track of stocks using digital technology in my hospital practice	0.88	4 (1)	4.25 ± 0.652	97 (88.2)	13 (11.8)
INT2	In my hospital practice, I intend to provide medicine to the wards using digital technology	0.88	4 (1)	4.23 ± 0.645	97 (88.2)	13 (11.8)
INT3	I intend to do patient education and counseling using digital technology in my hospital practice	0.85	4 (1)	4.16 ± 0.711	90 (81.8)	20 (18.2)
INT4	I intend to monitor and manage drug therapy using digital technology in my hospital practice	0.90	4 (1)	4.25 ± 0.612	100 (90.9)	10 (9.1)
INT5	I intend to reduce medication dose related errors by using digital technology in my hospital practice	0.90	4 (1)	4.30 ± 0.583	103 (93.6)	7 (6.4)
INT6	I intend to do therapeutic drug monitoring using digital technology in my hospital practice	0.85	4 (1)	4.19 ± 0.697	94 (85.5)	16 (14.5)
INT7	I intend to increase patient compliance by using digital technology in my hospital practice	0.80	4 (1)	4.15 ± 0.715	89 (80.9)	21 (19.1)
INT8	I intend to increase compliance with the guideline based on disease and drug management by using digital technology in my hospital practice	0.92	4 (1)	4.25 ± 0.623	99 (90.0)	11 (10.0)
INT9	I intend to identify disease-related risk factors using digital technology in my hospital practice	0.84	4 (1)	4.16 ± 0.684	92 (83.6)	18 (16.4)
INT10	I intend to reduce drug side effects by using digital technology in my hospital practice	0.84	4 (1)	4.23 ± 0.686	94 (85.5)	16 (14.5)
INT11	I intend to make digital technology remotely accessible (via phone, tablet and computer) in my hospital practice	0.81	4 (1)	4.19 ± 0.748	92 (83.6)	18 (16.4)

*ATT* attitude, *SN* subjective norm, *PBC* perceived behavioural control, *SE* self-efficacy, *PB* past behavior, *INT* intention For ATT, SN, PBC, SE and INT, positive/high frequency means the percentage of agreement (total of strongly agreed and agreed) and negative/low frequency means the percentage of disagreement (total of strongly disagree, disagree and neutral). For PB, positive/high frequency means the percentage of the total of regularly and often; negative/low frequency means the percentage of the total of never, occasionally and sometimes

The subscales and their descriptive statistics are shown in Table 2. Most of the participants thought that “The use of digital technology can increase compliance with the guidelines on disease and medication management” (95.5%), “Digital technology helps pharmacists to prevent drug errors and increase drug safety” (94.5%) and “Digital technology supports rational drug use and contributes to optimizing drug effectiveness” (94.5%). Compared to the other items in the attitude category, the number who thought that the use of digital technology could improve the quality of life of the patients was lower (80.9%).

In the subjective norm category, 79.1% and 82.7% of the participants stated that the chief pharmacist and their other pharmacist colleagues supported integrating digital technology into pharmacy services, respectively, and only 47% stated that the chief physician/manager supported it.

In self-efficacy, 103 (93.6%) participants thought they could easily avoid drug related problems using digital technology. Also, in past behaviours, it was determined that they used digital technology the most to prevent medication errors (n:71, 64.5) and the least in patient education and counseling (n:46, 41.8%).

In perceived behavioural control while 73 (66.4%) participants stated that they had the necessary skills and knowledge to use digital technology in pharmacy services, only 42 (38.2%) stated that they had sufficient hardware support (such as software) to use digital technology in pharmacy services.

In the responses to questions relating to intention, more than eighty percent of the participants said they intend to use digital technology in daily practice.

There was no statistically significant relationship between the intention to use digital technology in pharmacy service and their sex, educational status, years of experience, and having received training on digital technology (*p* > 0.05). Their intention was statistically related to their working place (*p* = 0.03), attitude (*p* < 0.001), subjective norm (*p* < 0.001), past behaviour (*p* < 0.001), self-efficacy (*p* < 0.001), and perceived behavioural control (*p* < 0.001). Table 3 presents multiple linear regression analyses that evaluate the factors associated with the total intention score. A positive attitude (*p* = 0.005), higher self-efficacy (*p* < 0.001), and employment in tertiary institutions (*p* = 0.017) had associations with the overall intention score (Table 3). For every 1-point increase in the attitude score, the intention score increased by 0.43 points, and for each 1-point increase in the self-efficacy score, the intention score increased by 1.34 points. The intention scores of pharmacists working in tertiary hospitals were 2.67 units higher than those working in secondary hospitals.

**Table 3:** Multiple linear regression analysis predicting hospital pharmacists’ intentions to use digital technology in their daily practice

Factors	Unstandardized coefficients		Standardized coefficients	
	B	Std. Error	Beta	*p* value
Constant	12.41	4.19		0.004
Attitude	0.43	0.12	0.28	0.005
Self-efficacy	1.34	0.36	0.37	< 0.001
Working place (Tertiary hospital)	2.67	1.10	0.19	0.017

*n* = 110, R = 0.621, R^2^ = 0.39 F = 22.22 *p* < 0.001

## Discussion

### Statement of key findings

The factors associated with the intentions of hospital pharmacists to use digital technology in their daily practice have been highlighted in this study. It was shown that hospital pharmacists’ positive attitudes and higher self-efficacy were associated with higher intention scores for using digital technology. Working in tertiary hospitals was another factor associated with their daily intention to use digital technology.

### Interpretation

Regarding attitude, nearly all pharmacists indicated that digital technology could improve adherence to disease and medication management guidelines, help pharmacists prevent drug errors, increase drug safety, support rational drug use, and optimize drug effectiveness. Although previous survey research has not investigated the attitude to intention relationship, reports are describing the utilization of digital technologies to supply clinical pharmacy services, including chronic disease management, medication review and health education, with pharmacists reporting favorable attitudes toward digital technologies [32–34]. The current study extends and supports these findings such that attitude was a significant and positive predictor of pharmacists’ high intentions.

Regarding subjective norm, while most participants stated that the chief pharmacist and their other pharmacist colleagues supported integrating digital technology into pharmacy services, only less than half said the chief physician/manager supported it. Cobelli et al. suggested that social influence does not affect a pharmacist’s intention to adopt telemedicine [35]. Similarly, our results show that the subjective norm was not associated with the use of digital technology. This may be because the use of digital technology in the hospital is thought to be especially related to the economic and social barriers, independent of the chief physician/manager, chief pharmacist, and other pharmacist colleagues. Especially in some countries, economic barriers prevent access to energy and the internet [36].

The results also show that self-efficacy was significantly associated with the intention to employ digital technology in daily practice, but perceived behavioural control was not. In contrast to our results, Gazava et al. found that perceived behavioural control was associated with intention to use a prescription drug monitoring program [37]. In addition, Darnell et al. reported that an educational conference series about digital health increased the self-confidence, self-efficacy and intention to use digital technology of pharmacy students [38]. Therefore, parameters that directly or indirectly affect self-efficacy also have an impact on the intention to use digital technology. Our prior research indicated a significant association between self-efficacy and the intention to deliver the pharmaceutical care [20]. The use of only two items to assess perceived behavioral control may have led to the non-significant findings in this study. Furthermore, this study operationalized the perceived behavioural control and self-efficacy construct as necessary and ease/difficulty, respectively. Other factors, such as controllability and facilitating conditions, were not measured, which could have altered the model’s importance of the construct. Further research is needed to assess the importance of perceived behavioral control and self-efficacy in the use of digital technology in daily practice.

The past behaviour construct, like perceived behavioural control and subjective norm in this study, was not significantly associated with the intention to use digital technology in daily practice and the use of digital technology was less than fifty percent of pharmacists. The primary explanation could be that nearly half of the pharmacists have no experience in providing patient education and counseling, lowering prescription costs, monitoring drug therapy, and enhancing medication adherence with digital technology. Similar to our study, 46% of pharmacists in the study by Hiskey et al. were using digital technology in pharmacy services, especially telehealth technology [39].

With the exception of workplace, no demographic variables were found to be associated with intention. In another study, Herbert et al. similarly reported that pharmacist characteristics were not significantly associated with intentions to provide Medicare medication therapy management services [40]. The intention scores of pharmacists working in tertiary hospitals were higher than those working in secondary care hospitals in our study. This may be due to the fact that people in management at the tertiary hospital have an academic background and may have had a more innovative approach to the application and hospital management system. As a result, pharmacists working such places may have had a greater opportunity to access and use digital technology.

### Strengths and weaknesses

There are some limitations to this study. First, it would be better if we conducted the study with more pharmacists; however, the sample size requirements were satisfied by the number of participants. The study only included pharmacists attending a specific congress. This may limit generalizability, as attendees may not represent all hospital pharmacists, particularly those who did not attend the congress. There is a need for more extensive studies involving many hospital pharmacists from rural and urban areas. The other limitation of the study was sample selection bias. Hospital pharmacists interested in digital technology might be willing to participate in the survey. These could impact the generalizability of study findings. Longitudinal studies can be conducted to draw causal information. The study included a broad category of digital technologies, but future studies could explore in more in-depth the adoption of specific technologies (e.g., mobile apps, digital drug management systems, artificial intelligence) to better understand the preferences, barriers, and facilitators related to each type of technology.

Despite the limitations, the study also has strengths. The study was based on the TPB, a widely recognized and established theoretical model in social psychology, which is highly relevant for understanding health professionals’ intentions and behaviours. This provides a solid foundation for analyzing participants’ digital health technology adoption. Also, by focusing on hospital pharmacists’ engagement with digital health systems, the study has practical implications for improving digital health initiatives and strategies, especially in the healthcare sector. Understanding the attitudes, behaviours, and factors influencing the adoption of digital health technologies can inform targeted interventions to enhance technology uptake.

### Further research

Understanding pharmacists’ perspectives is very important for optimizing service delivery. Policymakers allocating budget to this area will facilitate access to digital technology for use in pharmacy services. The implementation of technology-focused curricula in pharmacy education is essential for enhancing knowledge, ensuring the success of future pharmacists in the digitally advancing healthcare environment, enhancing their confidence in utilizing digital technology, and ultimately elevating their tendency to employ such technology. Addressing these many aspects will enable the pharmaceutical sector to adeptly navigate the problems and opportunities of the digital era, fostering the development of robust and innovative practice models in the future. Future research may investigate organizational or institutional barriers that could hinder or delay the incorporation of digital technologies into pharmacy services.

## Conclusion

The factors associated with the intentions of hospital pharmacists to use digital technology in their daily practice have been highlighted in this study. Hospital pharmacists’ positive attitudes and higher self-efficacy were associated with higher intention scores for using digital technology. Working in tertiary hospitals was also related to their daily intention to use digital technology. These factors can be considered while developing intervention studies to promote hospital pharmacists’ usage of digital technology in hospital settings.
